# Resurrection of a Bull by Cloning from Organs Frozen without Cryoprotectant in a −80°C Freezer for a Decade

**DOI:** 10.1371/journal.pone.0004142

**Published:** 2009-01-08

**Authors:** Yoichiro Hoshino, Noboru Hayashi, Shunji Taniguchi, Naohiko Kobayashi, Kenji Sakai, Tsuyoshi Otani, Akira Iritani, Kazuhiro Saeki

**Affiliations:** 1 Gifu Prefectural Livestock Research Institute, Takayama, Gifu, Japan; 2 Department of Genetic Engineering, Kinki University, Kinokawa Wakayama, Japan; 3 Wakayama Industry Promotion Foundation, Wakayama, Japan; Duke Unviersity, United States of America

## Abstract

Frozen animal tissues without cryoprotectant have been thought to be inappropriate for use as a nuclear donor for somatic cell nuclear transfer (SCNT). We report the cloning of a bull using cells retrieved from testicles that had been taken from a dead animal and frozen without cryoprotectant in a −80°C freezer for 10 years. We obtained live cells from defrosted pieces of the spermatic cords of frozen testicles. The cells proliferated actively in culture and were apparently normal. We transferred 16 SCNT embryos from these cells into 16 synchronized recipient animals. We obtained five pregnancies and four cloned calves developed to term. Our results indicate that complete genome sets are maintained in mammalian organs even after long-term frozen-storage without cryoprotectant, and that live clones can be produced from the recovered cells.

## Introduction

In several mammalian species, cloned animals have been successfully produced by somatic cell nuclear transfer (SCNT) [1]–[5]. The integrity of the genome in the donor nuclei is essential for the full-term development of such cloned animals. Therefore, somatic cells of endangered or extremely valuable animals have been cryopreserved for maintenance of genetic resources [6]–[8]. To maintain the viability of mammalian cells for long-term storage, the cells are usually frozen-stored with appropriate cryoprotectants [9]. However, in cases in which donor individuals are already dead or their species extinct, cryopreserved cells with intact nuclei are not always available. There have been attempts to rescue animal genetic resources from dead cells or non-cryoprotected frozen specimens. Freeze-dried spermatozoa [10] and nuclei of spermatids in frozen mouse testes without cryoprotectant [11], when injected into oocytes, could produce normal pups, indicating that genomic integrity was maintained in some dead male gametes after freezing. Moreover, production of viable SCNT offspring from dead [12] and heat-denatured [13] somatic cells suggested that live donor cells were not required for SCNT. Recently, Li and Mombaerts [14] established mouse nuclear transfer embryonic stem (ntES) cells from embryos cloned from nuclei of dead cells after freezing without cryoprotectant. Moreover, blastocyst development has been reported after nuclear transfer with freeze-dried somatic sheep cells [15]. However, such cells are usually severely damaged by ice crystals and osmotic stress [9]. Very recently, Wakayama et al. reported the generation of cloned mice from nuclei recovered from a body that had been frozen without cryoprotectant [16]. They showed that SCNT embryos from nuclei of frozen brain and tail blood could develop to term, although the developmental potential was very low for other frozen tissues [16]. Therefore it is unclear whether the nuclei in the other frozen tissues remained intact.

A bull named Yasufuku was one of the most important and famous sires in the history of breeding Wagyu cattle due to his contributions to the improvement of the quality of marbling, which is a major characteristic of Wagyu beef. Yasufuku died of senility at an age of 13.5 years in September 1993. His testicles were collected 12 hours after his death, then wrapped in aluminum foil and placed in a −80°C freezer without cryoprotectant for 10 years. The testicles were then transferred to liquid nitrogen without cryoprotectant for another 3 years. We examined whether intact and culturable somatic cells could be retrieved from the testicles and whether the nuclei from such cells could contribute to the development of viable offspring after SCNT with enucleated oocytes. In this study, we succeeded in obtaining four live cloned calves from these frozen organ cells. Three calves are healthy though one died two days after birth. To our knowledge, this is the first report of the resurrection of a dead elite livestock specimen from a non-cryoprotected frozen organ by cloning.

## Results

### Retrieving cells from bull testicles frozen without cryoprotectant for one to four months

Before experimenting with tissues from Yasufuku, we conducted some preliminary experiments with fresh frozen testicles. We collected testicles from three 12- to 15-month-old bulls and froze them at −80°C without any special treatment in a freezer for one to four months. We then dissected the frozen testicles into different parts, caput epididymis, cauda epididymis, spermatic cords and testes. The parts were thawed, minced and digested with collagenase and dispase and then cultured. In our preliminary experiments, we used Dulbecco's modified Eagle's medium (DMEM) or α-minimum essential medium (α-MEM) to obtain primary cultures from the frozen testicles. However, no cells grew from the thawed tissue. It was possible that cells in the thawed tissue might be with quite low proliferating activity even when they were alive. Therefore we selected MF-start™ that was developed to readily obtain initial outgrowth of cells with low proliferating activity at primary culture. We obtained live and culturable cells from both the caput epididymis and the spermatic cords but not from the cauda epididymis or the testes. Most of the culturable cells proliferated actively and populations expanded, suggesting that the cells were in normal condition. We used these cells to produce SCNT embryos by electrofusion with enucleated oocytes. Fibroblasts taken from bovine ear tissue were used as controls. The SCNT embryos were then cultured for 168 hours [17]. The SCNT experiments were repeated 3 times. The SCNT embryos reconstructed from cells derived from the frozen testicles did not differ significantly from SCNT embryos generated from control cells with regard to blastocyst rates from cultured embryos (22.1% and 20.2%, p>0.05, Student's t-test), number of cells in blastocysts [130±43 and 121±43 (mean±SD), p>0.05, Student's t-test)], or ratio of the number of cells in the inner cell mass relative to the total number of cells in the blastocysts (21.1% and 22.6%, p>0.05, Student's t-test). Our results indicated that culturable somatic cells could be retrieved from bovine organs that had been frozen without cryoprotectant and that the cells could be used for nuclear transfer.

### Retrieving cells from Yasufuku's testicles

**Figure 1:**
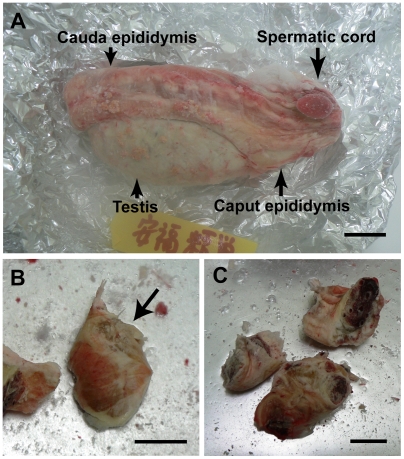
One of Yasufuku's testicles frozen for 13 years. The testicle was stored in a −80°C freezer for 10 years and then transferred to liquid nitrogen for 3 years. (A) Yasufuku's frozen testicle. (B) Part of the caput epididymis (arrow). (C) Spermatic cords that had been cut into three pieces. Scale bars represent 2 cm.

**Figure 2:**
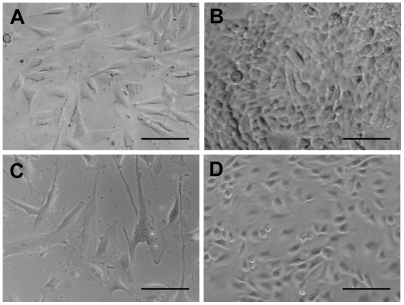
Differential interference-contrast micrograph of cell populations established from Yasufuku's frozen testicles. Cell lines A and B were used for SCNT after primary culture, and cell lines C and D were cryopreserved and then subcultured with five passages. (A) Cell line A, consisting of fibroblast-like cells. (B) Cell line B, consisting of epithelial-like cells. (C) Cell line C, consisting of fibroblast-like cells. (D) Cell line D, consisting of epithelial-like cells. Pregnancies were obtained from SCNT embryos that had been cloned from cells of line A (A) and line D (D). Scale bars represent 100 µm.

We isolated the caput epididymis and the spermatic cords from Yasufuku's frozen testicles and cut them into several small pieces (Fig. 1). We thawed, minced and digested the pieces of tissue as described above, and cultured the precipitates. We obtained four cultures of viable cells that were derived from two pieces of frozen tissue. As shown in Figure 2, the cells appeared to be either fibroblast-like (cell lines A and C; Figs. 2A and 2C, respectively) or epithelial-like (cell lines B and D; Figs. 2B and 2D, respectively). We used primary cultures of cell lines A and B for SCNT, and we cryopreserved cell lines C and D in a cell cryopreservation solution (Cellbanker; Mitsubishi Kagaku-Iatron, Tokyo, Japan), and then subcultured them with five passages. Cells of line C were enlarged and flattened, and their proliferative activity was reduced after freezing and thawing. However, cells of line D appeared to be normal and maintained proliferative activity even after freezing and thawing. A TUNEL assay (*In situ* cell death detection kit; Roche, Basel, Switzerland) revealed that most (71.4 to 75.0%) of the nuclei in cells of line C were apoptotic, while only a small percentage of the nuclei in cells of line D were apoptotic (1.3% to 5.4%), and the latter percentage did not differ significantly from that obtained with serum-starved control cells (4.1%, p<0.05, chi-square test). Moreover, chromosomal numbers were normal (60) in most (55%, 38/69) of the cells of line D.

### Cloning Yasufuku

**Figure 3:**
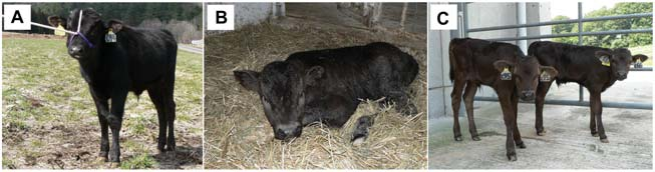
Calves cloned from Yasufuku's frozen testicles. (A) A male calf derived from Yasufuku's testicles was born on 30 November 2007. Parturition was induced by injection of prostaglandin F2α after 287 days of gestation and the recipient animal delivered this calf two days after induction. The calf's birth weight was 18.5 kg and he remains healthy at the time of writing. (B) A male calf, derived from a vitrified SCNT embryo, that was delivered by Caesarean section on 5 March 2008, after 286 days of gestation. The calf's birth weight was 47.5 kg; he died two days after birth. (C) Male calves derived from vitrified SCNT embryos. The calf with an ear tag “c95” was born on 22 July 2008, at 287 days of gestation. Its birth weight was 32 kg. The calf with an ear tag “c66” was born on 31 July 2008, at 288 days of gestation. Its birth weight was 30 kg. Parturitions were induced as described above. Both remain healthy at the time of writing.

We produced SCNT embryos by electrofusion of cells of three different cell lines (A, B and D) with enucleated bovine oocytes. SCNT blastocysts were produced from each cell line (Table 1). When we transferred six blastocysts that had been cloned from cell line A into recipient animals, three recipients became pregnant. Although one fetus was aborted due to mummification after six months, two developed to term and male calves were delivered. The first calf was born on 30 November 2007 and is still alive at the time of writing (Fig. 3A). The second calf from a vitrified SCNT embryo was born on 5 March 2008 (Fig. 3B), but he died after two days. When we transferred six blastocysts cloned from cell line D into six recipients, two recipients, which received vitrified embryos, delivered two healthy male calves on 22 and 31 July 2008 (Fig. 3C). DNA microsatellite analysis indicated that both the cloned calves and the aborted mummified fetus were derived from the cultured cells used as donors for SCNT, that the cells were derived from Yasufuku's testicles, and that the cells were different from those of the recipient animals (Tables 2 and 3).

**Table 1:** Development of embryos cloned from cells of three different Yasufuku cell lines.

Cell line	No. of experiments	No. of SCNT embryos	No. (%) of embryos fused*	No. of embryos cultured	No. (%) of blastocysts¶	No. of embryos transferred†	No. (%) of pregnancies‡	No. of live offspring
A	1	64	32 (50)	21	9 (43)	6	3 (50)^a^	2
B	1	62	21 (34)	21	7 (33)	4	0 (0)	-
D	6	289	242 (84)	162	27 (17)	6	2 (33)^b^	2
Subtotal	-	415	295 (71)	204	43 (21)	16	5 (31)	4
Control	6	289	226 (78)	106	41 (39)	-	-	-

* Fusion was examined one hour after electrofusion of couplets of cells and enucleated oocytes. Percentage of the number of the couplets.

¶ Blastocyst development was examined 168 h after nuclear transfer. Percentage of the number of cultured embryos.

† Single blastocysts were transferred into single synchronized recipients. Several blastocysts were stored in liquid nitrogen by a vitrification technique. Ten vitrified and warmed embryos were transferred to recipients.

‡ Pregnancy was examined by ultrasonography at 33 days after embryo transfer. Percentage of the number of transferred recipients.

a One recipient delivered a healthy male calf on 30 November 2007. One delivered a mummified fetus on 27 August 2007. One recipient that received a vitrified embryo delivered a healthy male calf on 5 March 2008 but the calf died two days after birth.

b Two recipients, which received vitrified embryos delivered two healthy male calves on 22 and 31 July 2008.

**Table 2:** DNA microsatellite analysis in 13 polymorphic loci^a^.

	BM1824	BM2113	ETH10	ETH225	ETH3	INRA023	SPS115
Yasufuku's frozen semen	180,188	135,137	274,274	145,147	117,117	198,208	246,256
Frozen testicles	180,188	135,137	274,274	145,147	117,117	198,208	246,256
Donor cultured cells	180,188	135,137	274,274	145,147	117,117	198,208	246,256
Mummified fetus	180,188	135,137	274,274	145,147	117,117	198,208	246,256
Recipient	180,**182**	**131**,135	274,274	145,**145**	**115**,117	198,208	246,256
Cloned offspring^1^	180,188	135,137	274,274	145,147	117,117	198,208	246,256
Recipient^1^	180,**180**	**123**,135	274,274	145,**145**	117,117	198,208	246,256
Cloned offspring^2^	180,188	135,137	274,274	145,147	117,117	198,208	246,256
Recipient^2^	180,**182**	**125**,137	**268**,274	145,**145**	117,117	**212**,**212**	**248**,**252**
Cloned offspring^3^	180,188	135,137	274,274	145,147	117,117	198,208	246,256
Recipient^3^	180,**180**	135,137	274,274	145,147	117,**123**	198,208	**252**,256
Cloned offspring^4^	180,188	135,137	274,274	145,147	117,117	198,208	246,256
Recipient^4^	**182**,188	**133**,135	**272**,274	**143**,147	115,117	198,**212**	**252**,256

	TGLA122	TGLA126	TGLA227	TGLA53	DIK023	DIK010
Yasufuku's frozen semen	148,150	111,117	77,93	157,167	90,100	185,195
Frozen testicles	148,150	111,117	77,93	157,167	90,100	185,195
Donor cultured cells	148,150	111,117	77,93	157,167	90,100	185,195
Mummified fetus	148,150	111,117	77,93	157,167	90,100	185,195
Recipient	148,150	111,117	77,**77**	**165**,**177**	90,100	185,195
Cloned offspring^1^	148,150	111,117	77,93	157,167	90,100	185,195
Recipient^1^	148,**148**	**117**,117	77,93	167,**177**	**100**,100	185,**193**
Cloned offspring^2^	148,150	111,117	77,93	157,167	90,100	185,195
Recipient^2^	148,148	111,117	77,93	157,**159**	**84**,90	185,**185**
Cloned offspring^3^	148,150	111,117	77,93	157,167	90,100	185,195
Recipient^3^	148,150	111,117	77,**77**	167,**177**	100,**104**	**193**,195
Cloned offspring^4^	148,150	111,117	77,93	157,167	90,100	185,195
Recipient^4^	**140**,150	111,117	77,93	**165**,167	**84**,100	185,195

a Polymorphism in 13 microsatellites was examined by the Maebashi Institute of Animal Science, Livestock Improvement Association of Japan, Inc (LIAJ).

Values different from those of donor cultured cells are indicated in boldface.

1, 2, 3, 4 Cloned calves born on 30 November 2007, 5 March, 22 and 31 July 2008, respectively.

The probability that the genotype of a non-cloned animal would completely match the donor cells at these 13 loci is less than 10^−12^.

**Table 3:** DNA microsatellite analysis in 18 polymorphic loci^a^.

	DIK069	DIK024	DIK102	DIK097	DIK106	DIK068	DIK039	DIK096	BM6026
Yasufuku's frozen semen	163,163	239,245	135,135	190,190	109,109	152,160	195,195	256,258	167,167
Donor cultured cells	163,163	239,245	135,135	190,190	109,109	152,160	195,195	256,258	167,167
Mummified fetus	163,163	239,245	135,135	190,190	109,109	152,160	195,195	256,258	167,167
Cloned offspring^1^	163,163	239,245	135,135	190,190	109,109	152,160	195,195	256,258	167,167
Cloned offspring^2^	163,163	239,245	135,135	190,190	109,109	152,160	195,195	256,258	167,167
Cloned offspring^3^	163,163	239,245	135,135	190,190	109,109	152,160	195,195	256,258	167,167
Cloned offspring^4^	163,163	239,245	135,135	190,190	109,109	152,160	195,195	256,258	167,167

	ETH185	ILSTS6	RM041	CYP21	BM4505	INRA130	BM103	ILSTS93	DIK093
Yasufuku's frozen semen	235,237	287,289	135,135	195,202	244,248	112,112	148,150	186,186	239,243
Donor cultured cells	235,237	287,289	135,135	195,202	244,248	112,112	148,150	186,186	239,243
Mummified fetus	235,237	287,289	135,135	195,202	244,248	112,112	148,150	186,186	239,243
Cloned offspring^1^	235,237	287,289	135,135	195,202	244,248	112,112	148,150	186,186	239,243
Cloned offspring^2^	235,237	287,289	135,135	195,202	244,248	112,112	148,150	186,186	239,243
Cloned offspring^3^	235,237	287,289	135,135	195,202	244,248	112,112	148,150	186,186	239,243
Cloned offspring^4^	235,237	287,289	135,135	195,202	244,248	112,112	148,150	186,186	239,243

a Polymorphism in 18 microsatellites were examined by our laboratory.

1, 2, 3, 4 Cloned calves born on 30 November 2007, 5 March, 22 and 31 July 2008, respectively.

## Discussion

In this study, we demonstrated that normal and actively proliferating cells can be retrieved from mammalian organs that have been frozen and stored without cryoprotectant for more than a decade and that normal healthy offspring can be produced from such cells by the nuclear transfer technique. To our knowledge, this is the first report of the cloning of a dead livestock specimen from unprotected frozen tissue. SCNT offspring have been produced from dead [12] and heat-denatured [13] somatic cells, indicating viability of donor cells is not required for SCNT. Therefore genomic integrity of donor cells is essential for the full-term development of cloned animals. Spermatozoa that were freeze-dried with cryoprotectant could maintain their genomic integrity but lost their motility [10]. Recently, blastocyst development has been reported after nuclear transfer with freeze-dried somatic sheep cells [15]. This finding suggests that genomic integrity of somatic cell nuclei may be maintained after freeze-drying. However, blastocyst development was obtained only when the cells were freeze-dried with a cryoprotectant (trehalose) [15]. Cells in organs or tissues that were frozen without a cryoprotectant are commonly thought to be extensively damaged. However, Ogonuki et al. found that virtually all spermatids in frozen mouse testes had extensively disintegrated cytoplasm or no cytoplasm around their nuclei, but that some of the spermatids, when injected into oocytes, could produce normal pups, indicating that they were genomically intact [11]. Recently, it was reported that mouse ntES cells were established from embryos cloned from nuclei of dead cells after freezing without cryoprotectant, and that chimeric mice were produced by the injection of the ntES cells into tetraploid blastocysts [14]. Very recently, Wakayama et al. generated cloned mice from ntES cells derived from nuclei from a body that was frozen without cryoprotectant for 16 years [16]. These results indicated that the nuclei of mouse somatic cells frozen even without cryoprotectant can be reprogrammed by SCNT. The combination of SCNT and ntES cell techniques may improve the probability of obtaining cloned animals from frozen tissues or organs in which the cells have been extensively damaged. In contrast to these reports, we obtained live, actively proliferating and normal cells from an organ that had been collected from a dead animal and frozen in a −80°C freezer for 10 years, and we produced four viable cloned cattle from five pregnancies by a conventional single-step SCNT technique. Our results indicate that small numbers of organ cells had withstood and/or evaded cryoinjury from ice crystals and/or osmotic stress within the organ and that nuclei in the cells were intact after defrosting the non-cryoprotected frozen organs. Our findings are consistent with the fact that frozen morselized human bone has been found to contain live cells [18]. Cells in certain tissues may be able to withstand cryoinjury, even when injury is caused by freezing without cryoprotectant. Spermatic cords consist mainly of fat tissue, blood vessels, nerve tissue, muscle tissue and connective tissue. We do not know which of these tissues, in the present study, had contained live cells after freezing. Further studies are needed to identify the organs or tissues from which live cells can be efficiently collected.

Gametes, zygotes, embryos and/or cell cultures derived from elite livestock or endangered animals have often been cryopreserved in ‘Gene Banks’ [7], [8]. Our results suggest that intact and living mammalian cells can be readily preserved by storing organs or tissues in a −80°C freezer without any special treatment. Therefore, it might be possible to rescue viable cells from frozen tissues or organs of dead animals or extinct species. Recently, clones have been successfully produced by cross-species nuclear transfer in several endangered species [12], [19], [20]. These results, together with our results suggest the possibility of restoring extinct species, such as woolly mammoths, if live cells can be retrieved from an organ or animal that has been frozen in a freezer or in the Siberian permafrost [21].

## Materials and Methods

### Freezing testicles

Testicles after castration were immediately wrapped in aluminum foil, and then frozen in a −80°C freezer without cryoprotectant. The testicles were stored in the freezer for one to four months. Yasufuku's testicles were taken from his scrota 12 hours after his death, then wrapped in aluminum foil, frozen in a −80°C freezer without cryoprotectant, Ten years later, his testicles were plunged into liquid nitrogen and stored for 3 years.

### Cell culture

Primary cultures of cells from thawed tissue were generated as described earlier [22]. Frozen tissue was thawed quickly by putting it into saline at 42°C. Thawed tissue was minced (∼5-mm in diameter) and incubated at 39°C for 2 h in Dulbecco's modified Eagle's medium (DMEM) that contained 0.1% collagenase (Invitrogen, Carlsbad, CA, USA) and 0.2% dispase (Invitrogen, Carlsbad, CA, USA) After filtration of the resultant suspension through 250-µm nylon mesh, the filtrate was centrifuged at 250×g for 5 min. Then the precipitate was resuspended in MF-start™ medium (Toyobo, Osaka Japan) and incubated at 38.5°C in an atmosphere of 5% CO_2_ in air with high humidity. After incubation for five days, the medium was then replaced with AmnioMAX™II complete medium (Invitrogen, Carlsbad, CA, USA) to induce the rapid proliferation of cells. Ten days later, after outgrowths had formed, the medium was replaced with MF-medium® (Toyobo, Osaka Japan).

### Nuclear transfer

SCNT by electrofusion of somatic cells with enucleated bovine oocytes was performed as described earlier [17]. The SCNT embryos were activated with 5 µM ionomycin for 5 min and then treated with 10 µg/ml cycloheximide in modified synthetic oviduct fluid medium (mSOFM) without KH_2_PO_4_ until 6 h post fusion (hpf) at 39°C in 5% CO_2_, 5% O_2_ and 90% N_2_ with high humidity. Following activation, the SCNT embryos were cultured in mSOFM until 168 hpf [17].

### Vitrification of cloned embryos

Some cloned embryos were vitrified prior to transfer to recipient animals as described elsewhere [23] with minor modifications. SCNT embryos were vitrified in 15% (v/v) ethylene glycol+15% (v/v) dimethylsulfoxide+0.6 M sucrose in tissue culture medium 199 supplemented with 20% fetal calf serum (TCM199) , using Cryotop (Kitazato BioPharma Co Ltd, Shizuoka, Japan) as a cryodevice. One embryo was placed on each Cryotop in a small volume of the vitrification solution (<1 µl). The Cryotop device was plunged into liquid nitrogen. After storage in liquid nitrogen, the embryos were thawed by immersing the Cryotop into 0.25 M sucrose in TCM199 for 1 min at 37°C. After washing 3 times, the embryos were transferred into recipient animals.

### Embryo transfer

Cloned blastocysts at 7 days post fusion were transferred into synchronized recipient animals non-surgically (one embryo per recipient) on day 7 or day 8 of the estrous cycle (estrus = Day 0) . All animal procedures in the present study were approved by the Committee for Experimental Animals of Gifu Prefectural Livestock Research Institute.

### DNA microsatellite analysis

Microsatellite analysis was performed, for parentage testing, for 13 microsatellites by the Maebashi Institute of Animal Science, Livestock Improvement Association of Japan, Inc. (LIAJ: Maebashi, Japan) as described elsewhere [2], [24]. Independently, we investigated an additional 18 microsatellites [25] to confirm the results. Moreover, additional 11 microsatellites were examined by LIAJ to confirm the results (data not shown).

## Supporting Information

Alternative Language Text S1 Japanese Translation of the Full Text by YH
(0.07 MB DOC)
