# Supplementary material for: Resurrection of a Bull by Cloning from Organs Frozen without Cryoprotectant in a −80°C Freezer for a Decade
Source: PLoS One. 2009 Jan 8;4(1):e4142. doi: 10.1371/journal.pone.0004142 (PMC2613553; doi:10.1371/journal.pone.0004142)
Supplement: Alternative Language Text S1 — Japanese Translation of the Full Text by YH (0.07 MB DOC) [file pone.0004142.s001.doc]

**Japanese translation**

**凍結保護剤を添加することなく-80℃の冷凍庫で10年間凍結された臓器からのクローン技術による種雄牛の復活**

星野 洋一郎*^‡^、林 登*^†‡^、谷口 俊仁^†‡^、小林 直彦*^†‡^、酒井 謙司*^†^、大谷 健*^†^、

入谷 明^†‡^、佐伯 和弘^†‡^

*岐阜県畜産研究所、^†^近畿大学生物理工学部遺伝子工学科、^‡^（財）わかやま産業振興財団

**要　約**

凍結保護剤を使用せずに凍結された動物組織の細胞は、体細胞核移植に用いる核ドナーとして不適当であると考えられていた。我々は、死んだ雄牛から採取された精巣を、凍結保護剤を使用せずに-80℃の冷凍庫で10年間凍結した後に、生きた細胞を取り出しクローン動物を作出することに成功したのでここに報告する。我々は凍結精巣の精索部分の組織片を解凍したところ、生きた細胞が回収できた。これらの細胞は、培養下で活発に増殖し、正常であると考えられた。我々はこれらの細胞で作製した16個の体細胞核移植胚を、16頭の仮親に移植した。その結果、5頭が受胎し、4頭のクローンの子牛が誕生した。我々の成果は、凍結保護剤を使用せずに長期間凍結保存された哺乳類臓器中の細胞に、完全なゲノムが保持されており、回収した細胞から生きたクローン個体を作出できることを明らかにした。

**緒　言**

何種類かの哺乳類において、体細胞核移植技術によるクローン動物が作製されている[1-5]。クローン動物が個体にまで発生するためには、ドナー核のゲノムが完全であることが必須である。そのため、絶滅危惧種や貴重な動物の遺伝資源を保存するために、それらの体細胞を凍結保存することが行われている[6-8]。これらの哺乳類細胞は、長期間の保存の間その生存性を保つために、ふつう、適切な凍結保護剤を用いて凍結保存されている[9]。しかし、ドナーとなる個体がすでに死んでいたり、その種が絶滅している場合、必ずしも完全な核を持つ細胞が凍結保存されて残っているとは限らない。死んだ細胞や、凍結保護されていない試料から動物遺伝資源を救出しようとする試みが行なわれている。マウスでは凍結乾燥した精子を卵子に注入することによって子供が生まれている[10]。あるいは、凍結保護剤を用いずに冷凍されていたマウスの精巣から精子細胞を取り出し、その核を卵子に注入することによって、正常な子供が生まれている[11]。これらの結果は、雄の配偶子は凍結されて死んだ状態にあっても、完全なゲノムを保持していることを示している。さらに、死んだ体細胞[12]や、熱変性させた体細胞[13]からクローン産子が誕生したことから、ドナー細胞の生存性は体細胞核移植に必要ではないことが示唆されている。最近、LiとMombaertsはマウスにおいて、凍結保護剤を用いず凍結して死んだ細胞からクローン胚を作り、核移植胚性幹細胞(ntES細胞)を樹立した[14]。さらに、凍結乾燥されたヒツジの体細胞を核移植したクローン胚が、胚盤胞期胚まで発生したことが報告されている[15]。しかし、通常このような凍結細胞は、氷晶形成や浸透圧ストレスによって大きなダメージを受けている[9]。ごく最近、Wakayamaらは、凍結保護剤を用いずに冷凍されたマウスの死体の組織から細胞核を取り出し、核移植によってクローンマウスが誕生したことを報告した[16]。Wakayamaらによると、凍結マウスの脳組織および尾部血液の細胞の核を用いた核移植胚がクローン個体まで発生したが、他の凍結組織を用いた場合の発生能力は非常に低かった[16]。したがって、他の凍結組織の中の核が完全であるかどうかは明らかになっていない。

種雄牛「安福」号は、和牛肉の主な特徴である脂肪交雑の品質改良に貢献したことにより、和牛育種の歴史において名高い重要な種雄牛のひとつであった。安福は1993年9月に13.5歳で老衰により死亡した。死後12時間後に精巣が取り出され、アルミホイルで包まれ、-80℃の冷凍庫で10年間冷凍保存された。その後、冷凍精巣は液体窒素の中に移され、さらに3年間、凍結保護剤を用いずに凍結された。我々は、この精巣から培養可能な生きた細胞を回収できるかどうかを調べた。そして、それらの細胞を除核卵子に核移植することによって、生きたクローン個体まで発生できるかどうかを調べた。その結果、凍結臓器の細胞から4頭のクローンの子牛を誕生させることに成功した。1頭は生後2日で死亡したがこれらの子牛は健康であり、異常は見られなかった。我々の知る限り、この研究は死亡した優良家畜を、凍結保護せずに凍結された臓器からクローン技術で再生することに成功した世界初の報告である。

**結　果**

**凍結保護剤を用いずに1-4ヶ月間凍結したウシ精巣からの細胞採取。**我々は安福の組織で実験する前に、新鮮なウシ凍結精巣を用いて予備実験を行った。12-15ヶ月齢の3頭の雄牛から精巣を採取し、なんら特別な処置を行わずに-80℃の冷凍庫で1-4ヶ月間凍結した。我々は凍結精巣を、精巣上体頭部、精巣上体尾部、精索組織、精巣組織の4つの異なる組織片に分割し、解凍した後、それぞれの組織を細切し、コラゲナーゼとディスパーゼによって消化培養を行い、回収された沈殿を培養した。この予備実験の前の実験において、我々は凍結精巣から初代培養細胞を得るためにダルベッコ改変イーグル培地（DMEM）とα最小必須培地（α-MEM）を用いたが、解凍した組織からは細胞が増殖しなかった。解凍した組織の中の細胞は、たとえ生きていたとしても増殖能力が極めて低い可能性が考えられた。そこで我々は、増殖能力の低い細胞から増殖を得るために開発された初代培養用培地MF-start^TM^（東洋紡ライフサイエンス）を用いた。その結果、我々は精巣上体頭部および精索組織から、生きた細胞を培養することに成功した。一方、精巣上体尾部と精巣組織からは細胞は得られなかった。これらの細胞の大部分は活発に分裂し、細胞数が増殖したので、細胞の状態は正常であると考えた。我々はこれらの細胞を除核卵子と電気融合することにより、核移植胚を作製して発生能力を検討した。牛の耳組織から採取された線維芽細胞を対照区のドナー細胞として用いた。体細胞核移植胚は168時間培養された[17]。実験は3回行われた。凍結精巣由来の細胞を用いて作製された核移植胚の発生能力を、対照区と比較したところ、胚盤胞期胚への発生率（22.1%と20.2%）、胚盤胞期胚の細胞数（130±43と121±43）、胚盤胞期胚における内部細胞塊の細胞数の割合（21.1%と22.6%）において有意な差は見られなかった。以上の結果から、凍結保護剤を用いずに凍結されたウシの臓器から培養可能な体細胞を回収することができ、それらを核移植に利用できることが示された。

**安福の精巣からの細胞の回収。**安福の凍結精巣から精巣上体頭部と精索組織を分離し、それらをいくつかに切り分けて凍結組織片とした（Fig. 1）。これらの組織片を前述のように解凍、細切、消化し、沈殿を培養した。その結果、2つの組織片から、4系統の生きた細胞が得られた。Figure 2に見られるように、線維芽細胞様細胞（細胞株AとC）と上皮細胞様細胞（細胞株BとD）が得られた。細胞株AとBの細胞は初代培養で体細胞核移植に用い、細胞株CとDの細胞は細胞凍結溶液（Cellbanker; Mitsubishi Kagaku-Iatron, Tokyo, Japan）を用いて凍結保存し、5代目まで継代培養した。C株の細胞は凍結融解後、細胞が肥大化し平らになり、増殖能力が低下した。しかし、D株の細胞は凍結融解後も外見は正常であり、増殖能力も維持していた。TUNEL法(*In situ* cell death detection kit; Roche, Basel, Switzerland)によって細胞のアポトーシスを調べたところ、C株の細胞の多く（71.4%から75.0%）はアポトーシスを起こしていることがわかった。一方D株の細胞のアポトーシス率はわずかであり（1.3%と5.4%）、この割合は血清飢餓培養を行った対照区の細胞のアポトーシス率と変わらなかった（4.1%）。さらに、D株の細胞の多くの（55%,38/69）細胞の染色体数は正常（60本）であった。

**安福のクローニング。**我々は3つの細胞株（A, B, D）の細胞をウシ除核卵子と電気融合させて体細胞核移植胚を作製した。その結果、それぞれの核移植胚から胚盤胞期胚が得られた（Table1）。A株の細胞から作製されたクローン胚を仮親に移植したところ、3頭の仮親が受胎した。１頭の胎児は6ヶ月後にミイラ化して流産したが、2頭は最後まで発生し、雄の子牛が誕生した。最初の子牛は2007年11月30日に生まれ、現在も生存している（Fig. 3A）。2番目の子牛は凍結された核移植胚を用い、2008年3月5日に生まれた（Fig. 3B）。しかし2日後に死亡した。D株の細胞から作製された6個の胚盤胞を6頭の仮親に移植したところ、2頭の仮親が受胎し、それぞれ2008年7月22日と31日に健康な雄の子牛が誕生した（Fig.3C）。DNAマイクロサテライト分析によって、これらのクローンの子牛およびミイラ化した胎児は、安福の精巣から得られたドナー細胞に由来するクローンであることが確認された（Tables 2, 3）。

**考　察**

　本研究において、我々は凍結保護剤を用いずに10年以上凍結保存されていた哺乳類の臓器から、活発に増殖する正常な細胞を取り出すことが可能であり、これらの細胞を用いて核移植技術によって正常なクローン個体を作出できることを示した。我々の知る限り、これは凍結保護されずに凍結されていた死んだ家畜個体の組織から、家畜個体をクローン再生した世界初の報告である。体細胞クローン個体は死んだ細胞[12]や熱変成した細胞[13]からも誕生していることから、ドナー細胞の生存性は体細胞核移植には必要ないと考えられている。したがって、クローン個体が発生するために必須であるのは、ドナー細胞のゲノムが完全であることである。凍結保護剤を用いて凍結乾燥した精子は、運動性は失われるが、ゲノムの完全性は保たれている[10]。最近、凍結乾燥したヒツジの細胞を用いた核移植胚が、胚盤胞期胚へ発生したことが報告されている[15]。この知見は、体細胞のゲノムは凍結乾燥した後でもその完全性が保たれていることを示唆している。しかし、この報告においては、凍結保護剤（トレハロース）を用いて凍結乾燥した場合にのみ、胚盤胞期への発生が観察された[15]。一般に、凍結保護剤を用いずに凍結された臓器や組織の中の細胞は、著しく破壊されていると考えられていた。Ogonukiらの報告によると、冷凍されたマウス精巣内のほとんど全ての精子細胞が、核の周りの細胞質が著しく破壊されているか、細胞質が失われている状態であった。しかし、いくつかの精細胞を卵子に注入したところ、正常な産子が生まれた。このことは精細胞の核のゲノムが完全であったことを示している[11]。最近、凍結保護剤を用いずに凍結したマウスの体細胞の核からクローン胚を作製し、ntES細胞が樹立されたことが報告された。さらにこれらのntES細胞を4倍体の胚盤胞期胚に注入することにより、キメラマウスが誕生した[14]。ごく最近、Wakayamaらは凍結保護剤を用いずに16年間凍結されていたマウスの死体から、細胞核を取り出してntES細胞を作り、さらにntES細胞をドナー細胞としてクローンマウスが誕生したことを報告した[16]。これらの結果は、凍結保護剤を用いずに凍結されたマウスの体細胞の核であっても、体細胞核移植によってリプログラミングされES細胞になりうることを示した。体細胞核移植技術とntES細胞技術を組み合わせて用いることにより、細胞が著しく破壊された凍結組織や臓器からクローン動物を得る効率が高められると考えられる。本報告では、これらの報告とは対照的に、死んだ動物から取り出された後、-80℃の冷凍庫で10年間冷凍されていた臓器から、活発に増殖する生きた正常な細胞を採取することに成功した。これらの細胞を用いることにより、我々は一般的な1段階の核移植法を用いてクローン胚を作製し、5頭の受胎例から、4頭の生きたクローンの子牛を誕生させることに成功した。我々の成果は、凍結保護せずに凍結された臓器であっても、それらの臓器を解凍した後に、少数の細胞は氷晶形成や浸透圧ストレスによる凍結障害に耐えて、あるいは凍結障害をすり抜けて生存し、それらの細胞の核は完全であることを明らかにした。我々の発見は、ヒトの移植用凍結骨細片の中に生きた細胞が発見されたという報告とも一致している[18]。特定の組織の中の細胞は、たとえ凍結保護剤を用いずに凍結された場合でも、凍結障害に耐えることができると考えられる。精索組織は脂肪組織、血管、神経組織、筋肉組織、結合組織などからなる。本研究では、これらの組織のどれが凍結後に生きた細胞を保持していたのかは明らかになっていない。生きた細胞を効率的に得られる臓器や組織が何であるかを特定するためには、さらに研究が必要である。

　優秀な家畜や絶滅危惧動物から得られる配偶子、受精卵、胚や培養細胞は、「遺伝子銀行」にしばしば凍結保存される [7,8]。我々の結果は、哺乳類の臓器や組織を、特別な処理を行うことなく-80℃の冷凍庫で凍結するだけで、完全な生きた細胞を保存できることを示唆している。また、死んだ動物や絶滅動物の組織や臓器が凍結されて残っていれば、生きた細胞を救出できる可能性がある。最近、いくつかの絶滅危惧種において、異種間核移植によるクローン個体の作出が成功している[12, 19, 20]。これらの研究成果を、我々の結果と組み合わせることにより、絶滅動物を復活できる可能性がある。冷凍庫の中、あるいはシベリアの永久凍土等の自然環境で凍結されている、動物の臓器や死体から生きた細胞を採取することができれば、例えばマンモスのような動物であってもクローン技術により復活させることができるかもしれない。

**材料と方法**

**精巣の凍結**

　精巣は、去勢後直ちにアルミホイルに包み、凍結保護剤を用いずに-80℃の冷凍庫で凍結した。精巣は１ヶ月から４ヶ月の間、冷凍庫の中に保管された。安福の精巣は死後12時間後に陰嚢から取り出され、アルミホイルで包まれ、凍結保護剤を用いずに-80℃の冷凍庫で凍結された。10年後、安福の精巣は液体窒素中に移され、さらに3年間保管された。

**細胞培養**

凍結組織からの細胞の初代培養は、前例に従って行なった[22]。凍結組織は42℃の生理食塩水に投入することにより、素早く解凍した。解凍された組織を細切し(5mm角以下)、0.1%のコラゲナーゼ（Invitrogen, Carlsbad, CA, USA）と0.2%のディスパーゼ（Invitrogen）を含むダルベッコ改変イーグル培地(DMEM)内で、39℃で2時間培養した。250μmのナイロンメッシュで消化液を濾過し、濾液を250Gで5分間遠心沈降した。そして沈殿をMF-start培地（Toyobo, Osaka, Japan）で懸濁し、38.5℃、5%のCO_2_と空気の気相、高湿度の培養器で培養した。5日の培養後、細胞の素早い増殖を促すため、培地をAmnioMAX^TM^II完全培地（Invitrogen）に交換した。培養10日後、細胞増殖が形成されてきたら、培地をMF-medium®（Toyobo）に交換した。

**核移植**

体細胞と除核卵子の電気融合による体細胞核移植は、前例に従って行なった[17]。体細胞核移植胚は、5μMのイオノマイシンで5分間培養し、その後10μg/mlのシクロヘキシミドを添加した、KH_2_PO_4_を含まない改変合成卵管培地（mSOFM）で融合から6時間培養することによって活性化を行った。培養環境は39℃、5%のCO_2_、5%のO_2_、90%のN_2_の気相の高湿度環境で行った。活性化処理後、体細胞核移植胚をmSOFMに移し、融合後168時間培養した。

**クローン胚のガラス化保存**

いくつかのクローン胚は、仮親に移植されるまで、既報[23]に少し改変を加えた方法で凍結を行った。体細胞核移植胚は、tissue culture medium 199に20%のウシ胎児血清を加えた培地（TCM199）に、15%のエチレングリコール、15%のジメチルスルホキシド、0.6Mのスクロースを添加した培地を凍結培地として、凍結装置にクライオトップ（Kitazato BioPharma Co Ltd, Shizuoka, Japan）を用いて凍結した。クライオトップ１本につき、１個の胚をごく少量の凍結培地（1μl以下）と共に載せ、クライオトップを液体窒素に投入することによって凍結した。液体窒素での保管した胚は、0.25Mのスクロースを添加し、37℃に加温したTCM199にクライオトップを１分間浸すことによって解凍した。胚は、解凍後3回洗浄した後に、仮親に移植された。

**胚移植**

クローンの胚盤胞期胚は、融合から7日目の胚を、発情開始から7～8日目に同期化した仮親に非外科的に移植した（１頭につき胚１個）。この研究における全ての動物の処置については、岐阜県畜産研究所実験動物委員会の許可を得て行われた。

**DNAマイクロサテライト解析**

親子判定のためのマイクロサテライト解析は、既報にならって行った[2, 24]。解析は家畜改良事業団（LIAJ）によって13のマイクロサテライトマーカーを用いて行われた。我々は独自にも18のマイクロサテライトマーカー[25]を加えて分析し、結果を補強した。さらに、LIAJでは11の補完マーカーによる解析も行われ、結果が補強された（データ示さず）。

**謝　辞**

　我々は本論文に重要かつ有益な助言をいただいたD. Sipp氏（理化学研究所　発生・再生科学総合研究センター、神戸）に謝意を表する。この研究の一部は、科学技術振興機構・和歌山県地域結集型共同研究事業のプロジェクトによって行われた。

**参考文献**

1. Wilmut I, Schnieke AE, McWhir J, Kind AJ, Campbell KH (1997) Viable offspring derived from fetal and adult mammalian cells. Nature 385: 810-813.

2. Kato Y, Tani T, Sotomaru Y, Kurokawa K, Kato J, et al. (1998) Eight calves cloned from somatic cells of a single adult. Science 282: 2095-2098.

3. Wakayama T, Perry AC, Zuccotti M, Johnson KR, Yanagimachi R (1998) Full-term development of mice from enucleated oocytes injected with cumulus cell nuclei. Nature 394: 369-374.

4. Onishi A, Iwamoto M, Akita T, Mikawa S, Takeda K, et al. (2000) Pig cloning by microinjection of fetal fibroblast nuclei. Science 289: 1188-1190.

5. Wilmut I, Beaujean N, de Sousa PA, Dinnyes A, King TJ, et al. (2002) Somatic cell nuclear transfer. Nature 419: 583-586.

6. Ryder OA, Benirschke K (1997) The potential use of "cloning" in the conservation effort. Zoo Biol 16: 295 - 371

7. Corley-Smith GE, Brandhorst BP (1999) Preservation of endangered species and populations: a role for genome banking, somatic cell cloning, and androgenesis? Mol Reprod Dev 53: 363-367.

8. Ryder OA, McLaren A, Brenner S, Zhang YP, Benirschke K (2000) DNA banks for endangered animal species. Science 288: 275-277.

9. Pegg DE (2007) Principles of Cryopreservation. In: Day JG, Stacey GN, editors. Cryopreservation and Freeze-Drying Protocols: Second Edition. Totowa, NJ USA: Humana. pp. 39-58.

10. Wakayama T, Yanagimachi R (1998) Development of normal mice from oocytes injected with freeze-dried spermatozoa. Nat Biotechnol 16: 639-641.

11. Ogonuki N, Mochida K, Miki H, Inoue K, Fray M, et al. (2006) Spermatozoa and spermatids retrieved from frozen reproductive organs or frozen whole bodies of male mice can produce normal offspring. Proc Natl Acad Sci USA 103: 13098-13103.

12. Loi P, Ptak G, Barboni B, Fulka J, Jr., Cappai P, et al. (2001) Genetic rescue of an endangered mammal by cross-species nuclear transfer using post-mortem somatic cells. Nat Biotechnol 19: 962-964.

13. Loi P, Clinton M, Barboni B, Fulka J, Jr., Cappai P, et al. (2002) Nuclei of nonviable ovine somatic cells develop into lambs after nuclear transplantation. Biol Reprod 67: 126-132.

14. Li J, Mombaerts P (2008) Nuclear transfer-mediated rescue of the nuclear genome of nonviable mouse cells frozen without cryoprotectant. Biol Reprod: DOI:10.1095/biolreprod.1108.069583.

15. Loi P, Matsukawa K, Ptak G, Clinton M, Fulka JJ, et al. (2008) Freeze-dried somatic cells direct embryonic development after nuclear transfer. PLoS ONE 3: e2978.

16. Wakayama S, Ohta H, Hikichi T, Mizutani E, Iwaki T, et al. (2008) Production of healthy cloned mice from bodies frozen at -20 ^o^C for 16 years. Proc Natl Acad Sci U S A 105: 17318-17322.

17. Kasamatsu A, Saeki K, Tamari T, Iwamoto D, Tatemizo A, et al. (2007) Timing and uniformity of embryonic gene activation affect subsequent pre-implantation development of cloned bovine embryos. J Reprod Dev 53: 623-629.

18. Heyligers IC, Klein-Nulend J (2005) Detection of living cells in non-processed but deep-frozen bone allografts. Cell Tissue Bank 6: 25-31.

19. Lanza RP, Cibelli JB, Diaz F, Moraes CT, Farin PW, et al. (2000) Cloning of an endangered species (*Bos gaurus*) using interspecies nuclear transfer. Cloning 2: 79-90.

20. Sansinena MJ, Hylan D, Hebert K, Denniston RS, Godke RA (2005) Banteng (*Bos javanicus*) embryos and pregnancies produced by interspecies nuclear transfer. Theriogenology 63: 1081-1091.

21. Miller W, Drautz DI, Ratan A, Pusey B, Qi J, et al. (2008) Sequencing the nuclear genome of the extinct woolly mammoth. Nature 456: 387-390.

22. Pollard JW (1997) Basic Cell Culture Protocols. In: Pollard JW, Walker JM, editors. Basic Cell Culture Protocols: Second Edition. Totowa, NJ USA: Humana Press. pp. 1-12.

23. Laowtammathron C, Lorthongpanich C, Ketudat-Cairns M, Hochi S, Parnpai R (2005) Factors affecting cryosurvival of nuclear-transferred bovine and swamp buffalo blastocysts: effects of hatching stage, linoleic acid-albumin in IVC medium and Ficoll supplementation to vitrification solution. Theriogenology 64: 1185-1196.

24. Ashworth D, Bishop M, Campbell K, Colman A, Kind A, et al. (1998) DNA microsatellite analysis of Dolly. Nature 394: 329.

25. Inoue-Murayama M, Hirano T, Watanabe T, Mizoshita K, Yamakuchi H, et al. (1997) Individual identification and paternity control of Japanese Black cattle based on microsatellite polymorphism. Anim Sci Technol 68: 443-449.

**図の説明**

**Figure 1　13年間凍結されていた安福の精巣の一つ。**精巣は-80℃の冷凍庫で10年間保存され、その後液体窒素中で3年間保存された。（A）凍結された安福の精巣。（B）精巣上体頭部の一部（矢印）。（C）3つに分割された精索組織。スケールバーは2cmを示す。

**Figure 2　安福の凍結精巣から樹立された細胞群の位相差顕微鏡像**。AとBの細胞株は初代培養で体細胞核移植に用いられた。CとDの細胞株は凍結保存され、5代目まで継代培養された。（A）細胞株A、線維芽細胞様細胞。（B）細胞株B、上皮細胞様細胞。（C）細胞株C、線維芽細胞様細胞。（D）細胞株D、上皮細胞様細胞。妊娠例は、細胞株A(A)とD(D)の細胞をクローニングして作製したSCNT胚から得られた。スケールバーは100μmを示す。

**Figure 3　安福の凍結精巣からクローニングされた子牛。**（A）2007年11月30日に誕生した、安福精巣由来の雄の子牛。妊娠287日目に仮親にプロスタグランジンF2αを投与し、分娩を誘起した。仮親は誘起から2日後に出産した。子牛の生時体重は18.5kgであり、論文を執筆している現時点で健康である。（B）凍結した体細胞核移植胚から生まれた雄の子牛。妊娠286日の2008年3月5日に帝王切開によって誕生した。生時体重は47.5kgであった。この子牛は出生から2日後に死亡した。（C）凍結した体細胞核移植胚から生まれた2頭の子牛。”c95”の耳標の子牛は妊娠287日の2008年7月22日に生まれ、生時体重は32kgであった。”c66”の耳標の子牛は妊娠288日の 2008年7月31日に生まれ、生時体重は30kgであった。分娩は上記と同様の方法で誘起した。論文を執筆している現時点で、2頭とも健康である。
